# *ZmRop1* participates in maize defense response to the damage of *Spodoptera frugiperda* larvae through mediating ROS and soluble phenol production

**DOI:** 10.1002/pld3.468

**Published:** 2022-12-15

**Authors:** Haoran Zhang, Zongwei Hu, Xincheng Luo, Yuxue Wang, Yi Wang, Ting Liu, Yi Zhang, Longyan Chu, Xiangping Wang, Yangya Zhen, Jianmin Zhang, Yonghao Yu

**Affiliations:** ^1^ College of Agriculture Yangtze University Jingzhou China; ^2^ Guangxi Key Laboratory of Biology for Crop Diseases and Insect Pests Nanning China; ^3^ College of Life Sciences Yangtze University Jingzhou China

**Keywords:** defense response, ROS production, *Spodoptera frugiperda*, total soluble phenol, *Zea mays*, *ZmRop1*

## Abstract

As plant‐specific molecular switches, Rho‐like GTPases (Rops) are vital for plant survival in response to biotic and abiotic stresses. However, their roles in plant defense response to phytophagous insect's damage are largely unknown. In this study, the expression levels of nine maize RAC family genes were analyzed after fall armyworm (FAW) larvae infestation. Among the analyzed genes, *ZmRop1* was specifically and highly expressed, and its role in maize response to FAW larvae damage was studied. The results showed that upon FAW larvae infestation, salicylic acid and methyl jasmonate treatment *ZmRop1* gene transcripts were all down‐regulated. However, upon mechanical injury, the expression level of *ZmRop1* was up‐regulated. Overexpression of *ZmRop1* gene in maize plants could improve maize plant resistance to FAW larvae damage. Conversely, silencing of *ZmRop1* increased maize plant susceptibility to FAW larvae damage. The analysis of the potential anti‐herbivore metabolites, showed that *ZmRop1* promoted the enzyme activities of catalase, peroxidase and the expression levels of *ZmCAT*, *ZmPOD*, *ZmRBOHA* and *ZmRBOHB*, thereby enhancing the reactive oxygen species (ROS) production, including the content of O^2−^ and H_2_O_2_. In addition, overexpression or silencing of *ZmRop1* could have influence on the content of the total soluble phenol through mediating the activity of polyphenol oxidase. In summary, the results illuminated our understanding of how *ZmRop1* participate in maize defense response to FAW larvae damage as a positive regulator through mediating ROS production and can be used as a reference for the green prevention and control of FAW larvae.

## 1 INTRODUCTION

Small GTPases (GTP)‐binding proteins widely exist in eukaryotes. They are structurally divided into five families including Ras homolog (RHO), Rat sarcoma (RAS), RAS‐related nuclear (RAN), Rat brain (RAB), and adenosine diphosphate (ADP) ribosylation factor (ARF) (Rocha et al., 2017; Takai et al., 2001; Wong et al., 2001). A recent study showed that RHO and RAS families function as signaling switches in all reported eukaryotes, whereas proteins in other families are primarily related to the regulation of vesicle and large molecule movement (Miao et al., 2018).

Rho‐like GTPases (Rops) belong to the plant‐specific RHO subfamily and are monomer G proteins with molecular weight between 21 and 24 kD (Gu et al., 2004). Rops are also named RACs (Ras‐related C3 botulinum toxin Substrates) because of their high sequence similarity with Rac GTPases. Therefore, Rops is also commonly referred to as plant Rop/RAC protein (Feiquelman et al., 2018). Because the first Rop gene was isolated from peas, many Rops have been identified in plant species, such as *Arabidopsis thaliana*, *Zea mays*, *Brassica napus*, *Vitis vinifera*, *Oryza sativa*, *Medicago truncatula*, *Nicotiana tabacum*, *Hevea brasiliensis*, and *Solanum lycopersicum* (Miao et al., 2018; Yang & Watson, 1993)). Based on their molecular structure and motif conservation, Rops can be classified into four groups (I–IV) (Feiquelman et al., 2018).

As plant‐specific molecular switches, Plant Rop proteins play important roles in plant growth and development and response to all types of biotic and abiotic stresses. Firstly, plant Rop proteins are involved in plant growth and development, such as root hair formation (Stanislas & Jaillais, 2019), petal shape (Ren et al., 2016), pollen tube growth (Gu et al., 2003) and response to auxin (Tao et al., 2002). Secondly, plant Rop proteins also play key roles in plant response to various abiotic stresses. For example, overexpression of banana gene *MaRop5g* can enhance the tolerance of bananas to salt stress and *NtRop1* transgenic tobacco plants are more susceptible to salt injury (Cao et al., 2008; Miao et al., 2018). In response to low temperatures Rop GTPase is expressed in postharvest loquat fruits thereby regulating fruit lignification (Jin et al., 2009). In *Arabidopsis*, *AtRop6* is involved in reactive oxygen species (ROS) signaling response to iron deficiency stress (Zhai et al., 2018). In addition, how plant Rop proteins respond to various biological stresses is also an interesting topic for researchers, especially the study of plant Rop proteins in response to plant diseases. For example, in the case of rice pathogen *Magnaporthe grisea*, *OsRac1* positively regulates rice resistance to blast, whereas *OsRac4* and *OsRac5* act as a negative regulator to blast resistance. *OsRac6* has very little regulation of blast resistance, and *OsRac3* and *OsRac7* are not involved in the defense response of rice to blast (Chen et al., 2010; Wang et al., 2018). More importantly, *OsRac1* also has influence on the grain size and yield of rice through regulating cell division (Zhang et al., 2019). The expression of *TaRac1* in wheat is induced rapidly and strongly after stripe rust infection. The overexpression of *TaRac1* in tobacco increases the content of lignin and the ratio of glucosinol to lignin, thus enhancing the resistance to tobacco black tibia and bacterial fusarium wilt. In contrast, T*aRac3* and *TaRac4* are not involved in lignin accumulation, and further experiments revealed that *TaRac3‐*overexpressing plants are more susceptible to two diseases and *TaRac4* has no effect on the resistance to two diseases, but can promote the root growth of tobacco seedlings (Ma et al., 2017). Silencing of *HvRacB* in barley reduces the establishment of fungal haustoria and increases powdery mildew resistance (Schultheiss et al., 2002), whereas stable expression of *HvRacB*, *HvRac1* and *HvRac3* increases susceptibility to powdery mildew (Pathuri et al., 2008). Compared with other studies on plant defense response to plant diseases, few studies have been reported on the role of plant Rop proteins in insect resistance (Qiu et al., 2016; Yang et al., 2018).

As an important food crop, the investigation on insect resistance of maize has received much attention by researchers. Excellent progress has been made in the study of Rop proteins in maize. At present, nine Rop genes have been identified in maize, namely *ZmRop*1‐*ZmRop9* (Cao et al., 2012). In the existing reports, *ZmRop1*, *ZmRop4* and *ZmRop9* have potential roles in plant‐pathogen interactions (Christensen et al., 2003). It has been reported that *ZmRop1* (also known as *ZmRacA*), *ZmRop2* (*ZmRacB*), *ZmRop3* (*ZmRacC*) and *ZmRop4* (*ZmRacD*) can induce the production of ROS in mammalian NIH 3T3 cells (Hassanain et al., 2000), and *ZmRop2* has a specific role in male gametophyte function (Arthur et al., 2003). However, the functions of *ZmRop*s are still unknown in most cases, specifically their roles in plant‐insect interactions. Therefore, in this study, the transcripts of nine genes from maize Rop family were analyzed, and the function and mechanism of *ZmRop1* against fall armyworm (FAW) (*S. frugiperda*) were determined. The results will significantly increase our understanding of the important role of *ZmRop1* protein in plant defense response to FAW and provide a foundation for future studies of Z*mRop*s.

## 2 MATERIALS AND METHODS

### 2.1 Materials and culture condition

Maize variety Zhengdan 958 (*Z. mays*) was used in this study. Maize plants were grown in plastic pots (500 ml) filled with a soil mix (the ratio of vermiculite and humus = 1:1) at 25°C under 14 h light and 10 h dark conditions. All plants were used at maize trefoil stage (V3). FAW was collected from maize experimental field at Yangtze University and eggs were placed in petri dishes with a diameter of 9 cm in an incubator at 25 ± 2°C (16 light/8 dark) until they hatched. After hatching, they were fed with fresh maize leaves till the third instar, and placed individually in petri dishes with a paintbrush for single‐head feeding to prevent cannibalism. FAW instars were determined by recording the molting times.

### 2.2 Phylogenetic analysis of ZmRop proteins

All the Rop protein sequences, including maize, rice, wheat and *Arabidopsis*, were obtained from NCBI (https://blast.ncbi.nlm.nih.gov/Blast.cgi). Lasergene 7.0 was used to characterize the Rop protein sequences. The sequences of the Rop proteins were aligned using DNAMAN. The phylogenetic tree of *ZmRop*s and other plant Rop homologs was constructed using MEGA 7.0 with the maximum likelihood method (bootstraps = 1,000).

### 2.3 Maize induction treatment under different conditions

Two third‐instar larvae of FAW were inoculated on the heart leaf and the second leaf at V3. Leaves were harvested at 6, 12, 24, and 48 h after FAW infestation, respectively, and immediately placed in liquid nitrogen. For salicylic acid (SA) and methyl jasmonate (MeJA) treatment, .5 mmol/L SA solution and .1 mmol/L MeJA solution were sprayed on the foliage of maize plants, leaf samples were harvested at 6, 12, 24, and 48 h after treatment, respectively. For mechanical injury treatment, a scissors was used to create wounds on leaves to simulate feeding of FAW larvae, and samples were collected at 6, 12, 24, and 48 h after injury, respectively.

### 2.4 Vector construction and vacuum *agrobacterium* infiltration co‐cultivation

The CDS of *ZmRop1* with Xba1 and BamH1 sites was ligated into the corresponding sites of the overexpression vector pBI121 containing Cauliflower mosaic virus (CaMV) 35S promoter to generate pBI121‐*ZmRop1*; Specific fragments without functional domains of *ZmRop1* with Xba1 and BamH1 sites were ligated into the VIGS system of tobacco rattle virus (pTRV2) vector to generate pTRV2‐*ZmRop1*. The *ZmRop1*‐overexpressing vector (pBI121‐*ZmRop1*), the negative control with empty (pBI121), pTRV2‐*ZmRop1* vector, empty pTRV2 and pTRV1 vector were transformed into *Agrobacterium tumefaciens* GV3101. pTRV2‐*ZmRop1* and pTRV1 were mixed together to generate *ZmRop1*‐silencing vector (TRV:*ZmRop1*). pTRV2 and pTRV1 were mixed to generate the negative control with empty (TRV:00).

Transformation of maize wounded germinating embryos was done through *Agrobacterium* vacuum infiltration‐mediated (Wang et al., 2007; Zhang et al., 2017). A single colony of *A. tumefaciens* containing the corresponding plasmid was inoculated into LB liquid medium containing 50 mg/ml kanamycin (Kan) and 50 mg/ml rifampicin (Rif), and cultured at 28°C for 220 rpm until OD_600_ = about .4. The *A. tumefaciens* cultures containing pTRV1 and pTRV2‐*ZmRop1* were mixed at a ratio of 1:1. Acetosyringone (19.62 mg/L), cysteine (400 mg/L) and Tween 20 (5 ml/L) were added into each bacterial solution, and left to sit at room temperature for 2 h before use. Maize seeds were surface‐sterilized in 75% (v/v) ethanol for 1 min and 2.5% sodium hypochlorite for 3 min, then rinsed five times with sterile water. The seeds were cultivated in sterile water at 30°C in darkness for 24 h until the emerging sprouts were about 3 mm long. The seed coats were cut with a surgical blade, and seed embryos were punctured with a needle to form a micro‐wound opening. The seed embryos were completely immersed in *Agrobacterium* solution containing the corresponding vector under vacuum‐assisted infiltration for 5 min. The resulting preparations were co‐cultivated for 12 h in a shaker at 28°C, 180 rpm in flasks. The seed embryos were rinsed with sterilized water and placed on MS medium containing 5 mg/ml Kan. The seedlings were sown in sterilized soil after rooting. At the three‐leaf stage, leaves were harvested to determine the transformation effect by quantifying the expression level of *ZmRop1* (Figure S2). Untreated wild type (WT) maize seedlings and infected seedlings of bacterial liquid containing empty plasmid (pBI121 or TRV:00) were used as a control.

### 2.5 The resistance of *ZmRop1*‐overexpressing plants and *ZmRop1*‐silencing maize plants against FAW

Six leaves of the same size and length, two leaves from pBI121‐*ZmRop1* maize plants, pBI121 maize plants and wild type maize plants, were symmetrically placed on the inner edge of the petri dishe with a diameter of 15 cm, and ten larvae of the 1st instar, 2nd instar or 3rd instar of FAW larvae were placed at the center of a petri dish and counted after 2 h. Each treatment was replicated 10 times. The weight of newly hatched instar larva was measured in groups of 10, then placed in a petri dish with a diameter of 9 cm and provided with sufficient amount of leaves from pBI121‐*ZmRop1* maize plants, pBI121 maize plants and wild type maize plants for feeding, respectively. When the 1st instar larva of FAW became the 3rd instar larva and had fed for 60 h, the weight of 10 larvae was weighed as a group again. The survival rate of each instar larvae was calculated by subtracting the number of dead larvae from the number of tested larvae and dividing by the number of tested larvae. pBI121‐*ZmRop1*, pBI121 and WT maize plants with the same growth were put in a glass incubator. When FAW adults emerged for 1 day, one male and one female were chosen and put in a glass incubator and the number of FAW eggs was counted after 3 days. Five independent replicates were set up in each experiment. Similarly, The effects of TRV:*ZmRop1* maize plants, TRV:00 maize plants and WT maize plants against the changes of larva weight, the larva feeding choice and the adult laying egg choice of FAW were analyzed.

### 2.6 Determination of reactive oxygen content

The content of reactive oxygen in maize plants overexpressing or silencing *ZmRop1* was determined by nitroblue tetrazolium (NBT) staining (Han et al., 2022). The same parts of maize plants after different treatments were cut in Petri dishes, stained with NBT solution and exposed to light for 1 h, then decolorized with 90% ethanol solution in a water bath at 65°C for 10–15 min, and the leaves were observed. Superoxide anion (O^2−^) was determined using the hydroxylamine hydrochloride oxidation method (Fernandes et al., 2019). Leaves of each treatment were ground and dissolved in phosphate buffered saline (PBS) (PH = 7.8) to make an enzyme solution, and 10 mmol/L hydroxylamine hydrochloride was added to the mix. After 1 h, 17 mmol/L p‐aminobenzene sulfonic acid solution and 7 mmol/L a‐naphthylamine solution were added, and the supernatant was taken 20 min later, absorbance value of the mixture was measured at 530 nm. hydrogen peroxide (H_2_O_2_) was determined by iodimetry (Fernandes et al., 2019). The leaves of each treatment were ground and dissolved in .1% trichloroacetic acid (TCA). The supernatant was added to PBS (PH = 7.0) and 1 mol/L KI solution. After mixing, the absorbance value of the mixture was measured at 390 nm.

### 2.7 Measurement of the total soluble phenol

Maize leaves were put in a pre‐cooled mortar and ground to powder in liquid nitrogen. The powder (.1 g) was put in a 2 ml centrifuge tube, and 1.5 ml 80% methanol was added into the Eppendorf tube wrapped in tin foil to prevent photo‐oxidation. The supernatant was stirred on a vibrating screen at 150 rpm at 25°C overnight, then the supernatant (150 μl) was added to 150 μl 1 N Folin‐phenol reagent, shook well, and kept at room temperature for 5 min; 200 μl of 1 mol/L Na_2_CO_3_ solution was added, it was shaken well and kept for 10 min. Finally, 1 ml of double steamed water was added, shook well, and kept in the dark for 1 h. The absorbance value of the mixture was measured at 725 nm (Abdel Latef & Tran, 2016).

### 2.8 RNA extraction and qPCR analysis

Total RNA was extracted from maize roots, leaves, and stems using Trizol (invitrogen, China) according to the manufacturer's instructions. The quality of RNA was quantified using a NanoPhotometer N50 (Implen); 2 μg total RNA of each sample was reverse transcribed into cDNA using the PrimeScript™RT Reagent Kit (TaKaRa, Japan) according to the manufacturer's instructions. The cDNA used as a template for quantitative real‐time PCR (qPCR) with reverse transcriptase (Promega, USA). Maize Ubiquitin (*ZmUBI*) was used as the internal standard to normalize the variations in cDNA concentrations. The relative expression level of each gene was calculated using the 2^−ΔΔCt^ method (Livak & Schmittgen, 2001) and relative values of the expression level of each gene were shown as mean values of three independent tests, and three replicates performed for each independent test. All the primer sequences are shown in Table S1.

### 2.9 Statistical analysis

The statistical analyses were performed using SPSS v26.0 software. The differences in *ZmRop*s expression levels under different treatment was statistically analyzed using t‐test for independent samples. Two significance levels were used (*p < .05 and **p < .01). For the rest of the data one‐way analysis of variance (ANOVA) and Tukey's honestly significant difference (HSD) were performed to determine the significance of the data, the results of the analyses were considered significant at *p* < .05.

## 3 RESULTS

### 3.1 Sequence analysis of maize Rop/RAC proteins

**FIGURE 1:**
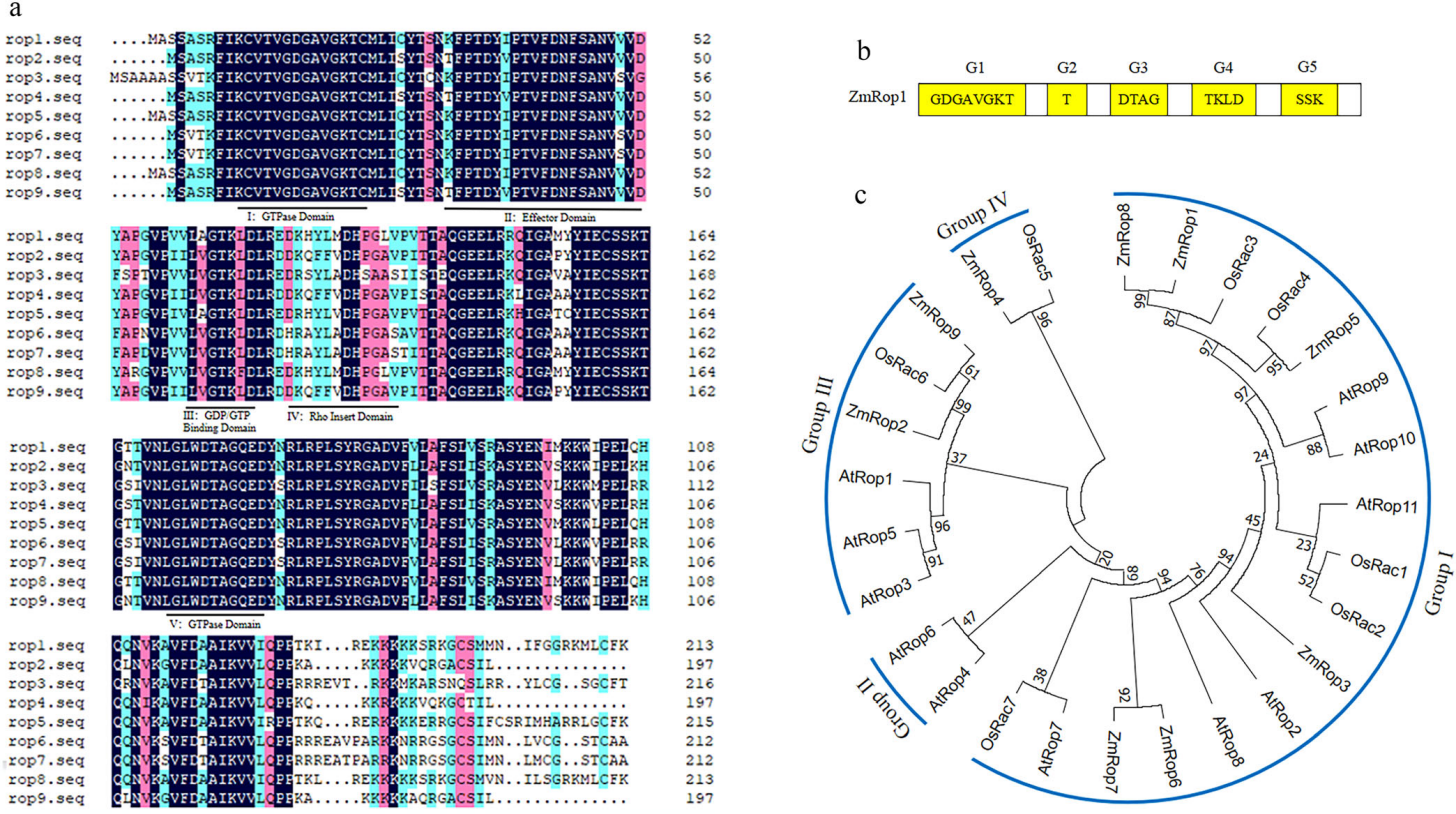
Alignment and phylogenetic analysis of *ZmRop*s. (a) Amino acid sequence alignment of *ZmRop*s. (b) Schematic representation of *ZmRop1*. G1‐G5 indicate conserved G motifs of *ZmRop*1. (c) Molecular phylogenetic tree for Rop proteins from *Arabidopsis thaliana* , *Oryza sativa* , *Triticum aestivum* and *Zea mays* . GenBank and EST accession numbers for these sequences are as follows: *Arabidopsis thaliana* *AtRop1* (U49971), *AtRop2* (U49972), *AtRop3* (AF115466), *AtRop4* (AF115472), *AtRop5* (AF115473), *AtRop6* (AF115470), *AtRop7* (AF115469); *Oryza sativa* *OsRac1* (AB029508), *OsRac2* (AB029509), *OsRac3* (AB029510), *OsRac4* (AK061102), *OsRac5* (AK067504), *OsRac6* (AK100842), *OsRac7* (AK058414); *Triticum aestivum* *TaRac1* (TC311608), *TaRac2* (TC321975), *TaRac3*, (TC293244), *TaRac4* (TC321480), *TaRac5* (TC316286), *TaRac6* (TC305987); and *Zea mays* *ZmRop1* (NP.001104929.1), *ZmRop2* (NP.001105615.1), *ZmRop3* (NP.001104930.2), *ZmRop4* (NP_001105719.2), *ZmRop5* (CAB96794.1), *ZmRop6* (CAB96793.1), *ZmRop7* (CAB96792.1), *ZmRop8* (AAK53059.1), and *ZmRop9* (NP.001105197.1).

The maize Rop/RAC family includes nine members (*ZmRop*s). Bioinformatic analysis revealed that proteins of *ZmRops (ZmRop1*‐*ZmRop9*) consist of 197 to 216 amino acids. Alignment analysis indicated that there is a higher similarity among the protein sequences of *ZmRop*s. The predicted protein secondary structure by SMART (http://smart.embl.de/) showed that all *ZmRop*s consisted of a GTP/Mg^2+^ binding site, a GTP hydrolase binding site (GAP), a guanine nucleotide exchange factor binding site (GEF), a guanine nucleotide dissociation inhibitor binding site (GDI), an effector interaction site, two GDP/GTP binding site (Switch region) and the characteristic conserved sequences of Small GTPases, five G‐boxes (Figure 1a,b). G2 overlaps with the Switch I region, also known as the effector region. G3 box overlaps with the Switch II region, which includes the Walker B motif (Figure S1). Phylogenetic tree of *ZmRop*s and Rops proteins from *A. thaliana*, *O. sativa* and *Z. mays* revealed that *ZmRop*s are classified into four groups. The largest group being group I which consist of *ZmRop5*, ZmRop1, ZmRop8, ZmRop6, ZmRop3 and ZmRop7, group II does not include any ZmRops, group III consist of ZmRop2 and ZmRop9, and group IV consist of ZmRop4 only (Figure 1c). This suggests that proteins in the same group are more likely to have similar functions.

### 3.2 The expression levels of *ZmRop1* are inhibited by the feeding of FAW larvae

**FIGURE 2:**
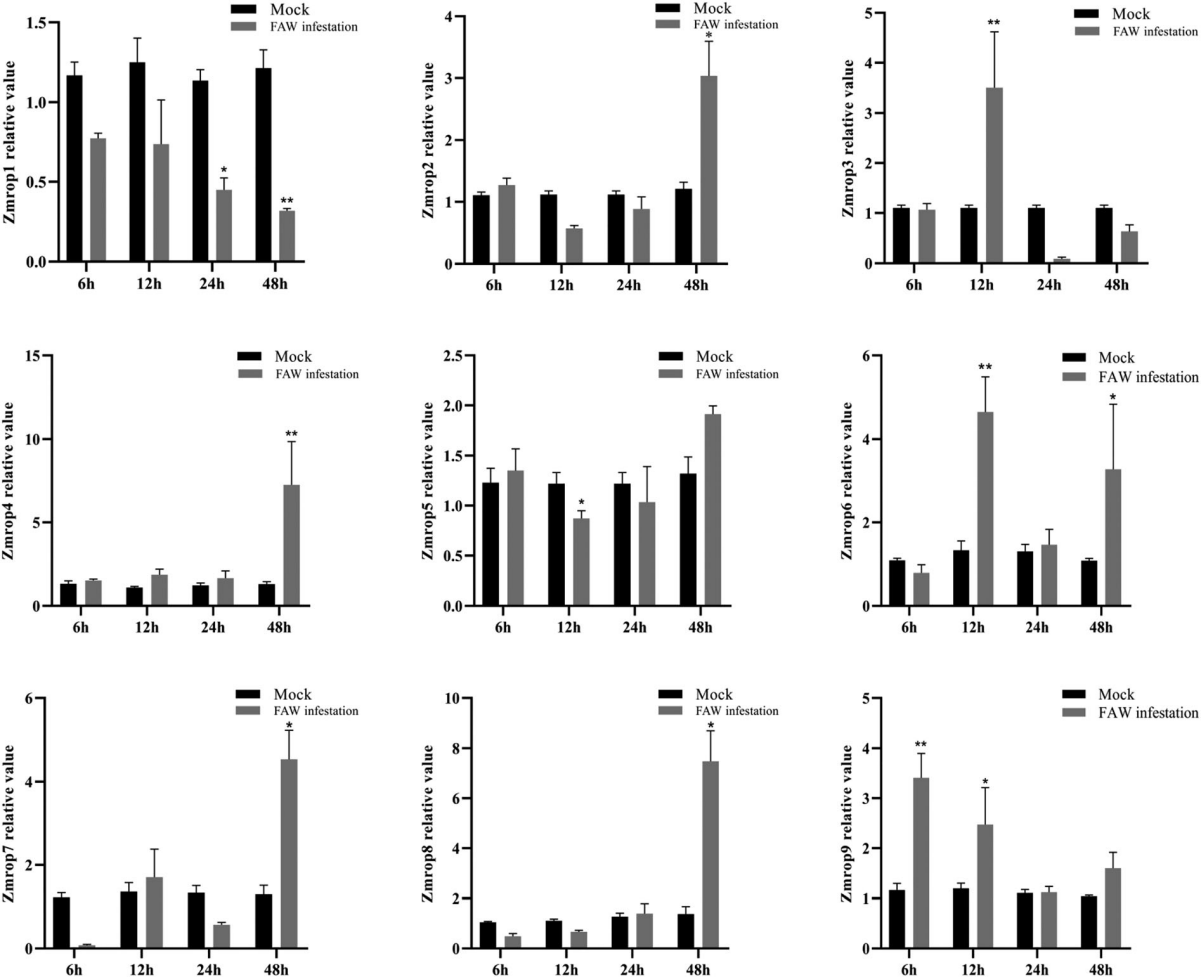
Expression levels of nine *ZmRop*s in maize leaves at different times under FAW infestation. Mean values and SD bars are provided (*p < .05, **p < .01; Student's *t*‐test).

FAW feeding induced expression levels of *ZmRops* to a different extent (Figure 2). The expression level of *ZmRop1* was downregulated at 6, 12, 24 and 48 h after FAW larvae feeding. Upon FAW larvae infestation *ZmRop1* was downregulated significantly after 24 and very significant after 48 h of feeding. The transcripts of *ZmRop2*, *ZmRop4* and *ZmRop8* were significantly upregulated at 48 h after FAW larvae feeding. The expression level of *ZmRop3* was very significantly upregulated at 12 h after FAW larvae feeding. At 12 h after FAW larvae feeding the expression level of *ZmRop5* significantly downregulated. The expression level of *ZmRop6* was significantly upregulated at 12 and 48 h under FAW larvae feeding. The expression level of *ZmRop9* was significantly upregulated at 6 and 12 h under FAW larvae infestation. The expression level of *ZmRop2* was significantly upregulated at 48 h, and *ZmRop7* expression was down‐regulated at 6 h but significantly upregulated at 48 h under FAW larvae feeding (Figure 2). All the results showed that *ZmRop1* was a differentially expressed gene, and its expression was inhibited by the feeding of FAW larvae.

### 3.3 The *ZmRop1* transcripts are regulated in abiotic stresses

**FIGURE 3:**
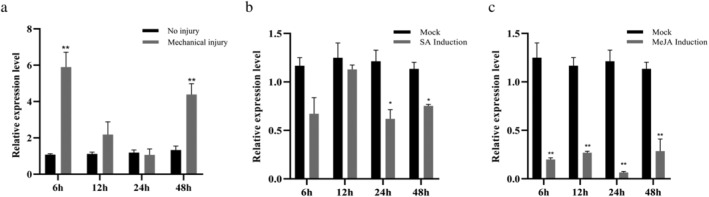
Expression levels of *ZmRop1* in maize leaves at different time points under SA, MeJA treatment and mechanical injury. (a) Mechanical injury. (b) SA treatment. (c) MeJA treatment. Mean values and SD bars are provided (*p < .05, **p < .01; Student's *t*‐test).

The transcripts of *ZmRop1* in maize leaves were quantified after the stimulation of mechanical injury, MeJA and SA treatment using qPCR. The results showed that the expression level of *ZmRop1* in maize leaves mostly showed an upward trend, and reached a very significant level at 6 and 48 h under the simulation of mechanical injury (Figure 3a). However, the expression level of *ZmRop1* in maize leaves was significantly low at 24 and 48 h after SA treatment (Figure 3b). In addition, under MeJA treatment, the expression level of *ZmRop1* was very significantly down‐regulated in maize leaves at different times (Figure 3c). These results suggested that mechanical injury could induce the expression of *ZmRop1*. It may also be affected by SA and JA signaling pathways.

### 3.4 *ZmRop1* overexpression enhances maize plant tolerance to FAW

**FIGURE 4:**
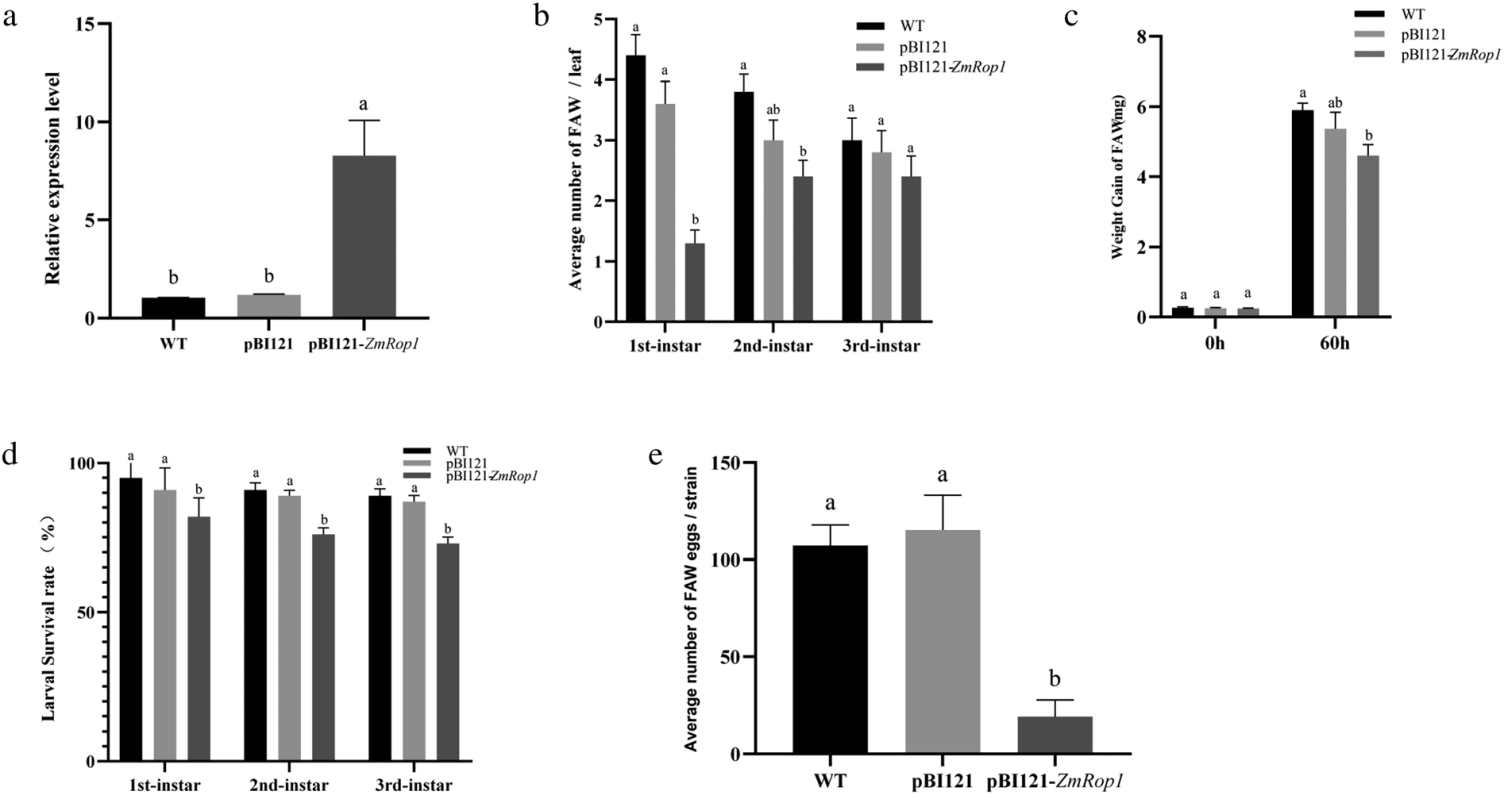
*ZmRop1* overexpression enhances maize plant tolerance to FAW. (a) Expression levels of *ZmRop1* in pBI121‐*ZmRop1*, pBI121 and WT maize plants. (b) The choice test of FAW larvae at different instars on pBI121‐*ZmRop1*, pBI121 and WT maize leaves. (c) Weight gain of FAW larvae at 60 h after feeding pBI121‐*ZmRop1*, pBI121 and WT maize leaves. (d) The survival rate of FAW larvae at different instars feeding pBI121‐*ZmRop1*, pBI121 and WT maize leaves. (e) The number of eggs of FAW adult on pBI121‐*ZmRop1*, on pBI121 and wildtype maize leaves. Different letters indicate significant differences (*p* < .05) based on Tukey's HSD test.

The *ZmRop1* transcript in pBI121‐*ZmRop1* maize plants was determined using qPCR. The results showed the expression level of *ZmRop1* in pBI121‐*ZmRop1* maize plants was significantly higher than that in pBI121 and WT maize plants, suggesting that *ZmRop1* expression level in pBI121‐*ZmRop1* maize plants was significantly up‐regulated (Figure 4a). The choice assay showed that the number of 1st instar larvae of FAW on pBI121‐*ZmRop1* maize leaves was significantly less than that on pBI121 and WT maize leaves. The number of 2nd instar larvae of FAW on pBI121‐*ZmRop1* maize leaves was lower than that on pBI121 and WT maize leaves, but there was a significant difference between the number of 2nd instar larvae of FAW feeding on pBI121‐*ZmRop1* maize leaves and WT maize leaves. There was no significant difference on the number of 3rd instar larvae of FAW feeding on WT, pBI121, and pBI121‐*ZmRop1* maize leaves (Figure 4b). Secondly, the no‐choice assay demonstrated that the weight gain of FAW larvae that had been feeding on pBI121‐*ZmRop1* maize leaves g for 60 h was significantly lower than FAW larvae feeding on WT maize leaves, but there was no significance difference when compared with that on pBI121 maize leaves (Figure 4c). Thirdly, when pBI121‐*ZmRop1* maize leaves were continuously fed, the survival rate of the first, second and third instar larvae were significantly lower than that on pBI121 and WT maize leaves (Figure 4d). Finally, the number of eggs laid by the larvae that fed on pBI121‐ *ZmRop1* maize leaves was significantly low compared with the number of eggs laid by larvae that fed on pBI121 and WT maize leaves (Figure 4e). These results indicated that *ZmRop1* overexpression enhanced maize plant tolerance to FAW larvae.

### 3.5 Silencing of *ZmRop1* increases maize plant susceptibility to *S. frugiperda*

**FIGURE 5:**
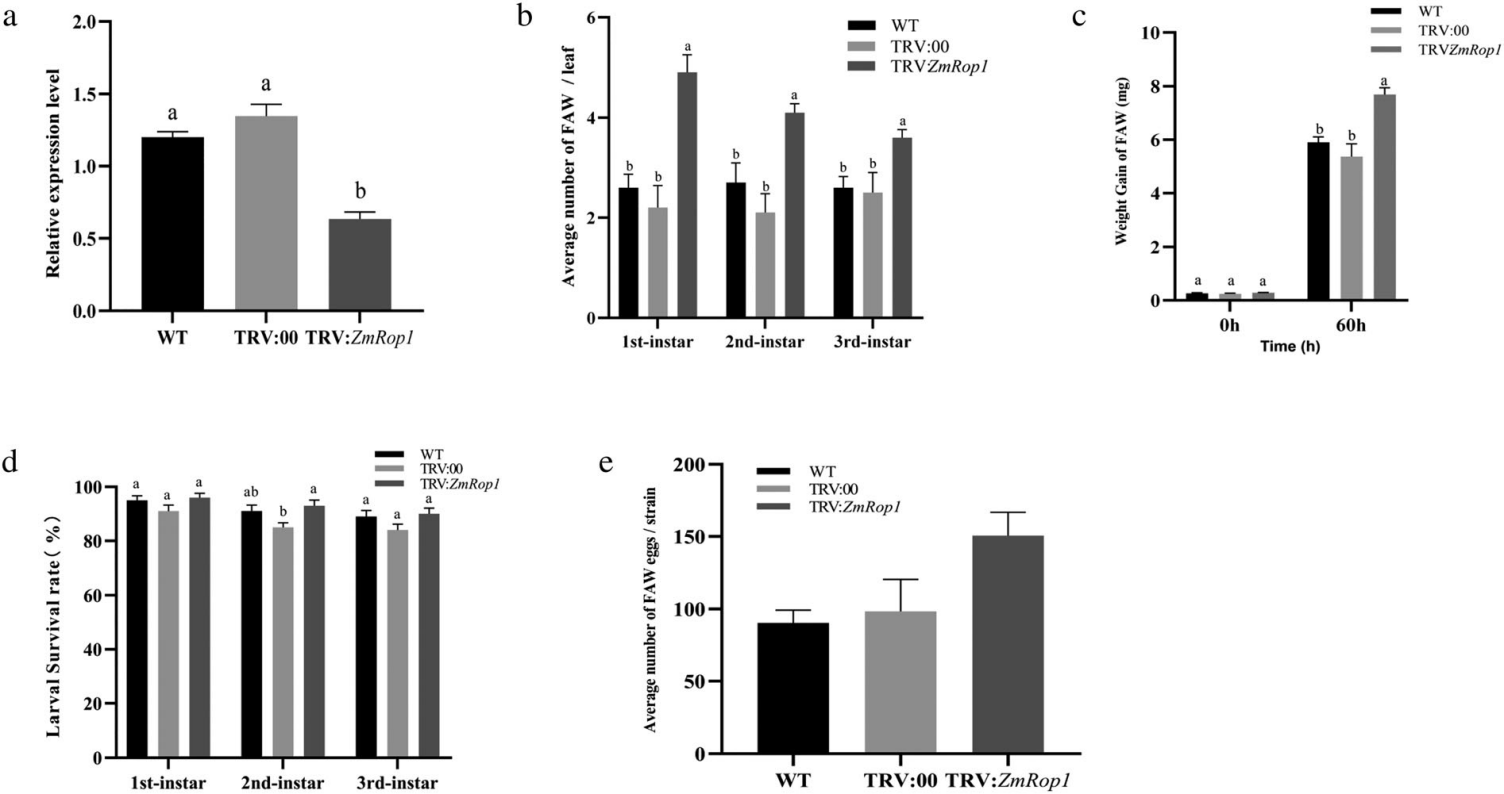
*ZmRop1* silencing decreases maize plant tolerance to FAW larvae. (a) Expression levels of *ZmRop1* in TRV:*ZmRop1*, TRV:00 and WT maize plants. (b) The choice of the larvae of FAW at different instars on TRV:*ZmRop1*, TRV:00 and WT maize leaves. (c) Weight gain of FAW larvae at 60 h after feeding TRV:*ZmRop1*, TRV:00 and WT maize leaves. (d) The survival rate of FAW larvae at different instars feeding TRV:*ZmRop1*, TRV:00 and WT maize leaves. (e) The egg number of FAW adult on TRV:*ZmRop1*, TRV:00 and WT maize leaves. Different letters indicate significant differences (*p* < .05) based on Tukey's HSD test.

Virus‐induced gene silencing (VIGS) was also used to elucidate the role of *ZmRop1* in maize response to FAW larvae damage. Compared with the WT and TRV:00 maize plants, the expression level of *ZmRop1* was significantly reduced in TRV:*ZmRop1* maize plants after *A. tumefaciens* infection (Figure 5a). The choice assay confirmed that at different instars the larvae of FAW preferred to feed on TRV:*ZmRop1* maize plants in contrast with TRV:00 and WT maize leaves (Figure 5b). Secondly, weight gain of FAW larvae at 60 h after feeding on TRV:*ZmRop1* maize leaves was significantly higher than those feeding TRV:00 and WT maize leaves (Figure 5c). However, after continuous feeding of TRV:*ZmRop1*, TRV:00 and WT maize leaves, there was no significant difference in the survival rate of FAW larvae at different instars (Figure 5d). Furthermore, the egg number of FAW adults on TRV:*ZmRop1* was higher than that on TRV:00 and WT maize leaves, but there was no significant difference among them (Figure 5e). These results revealed that silencing of *ZmRop1* increased maize plant susceptibility to FAW larvae.

### 3.6 *ZmRop1* expression influences the generation of ROS

**FIGURE 6:**
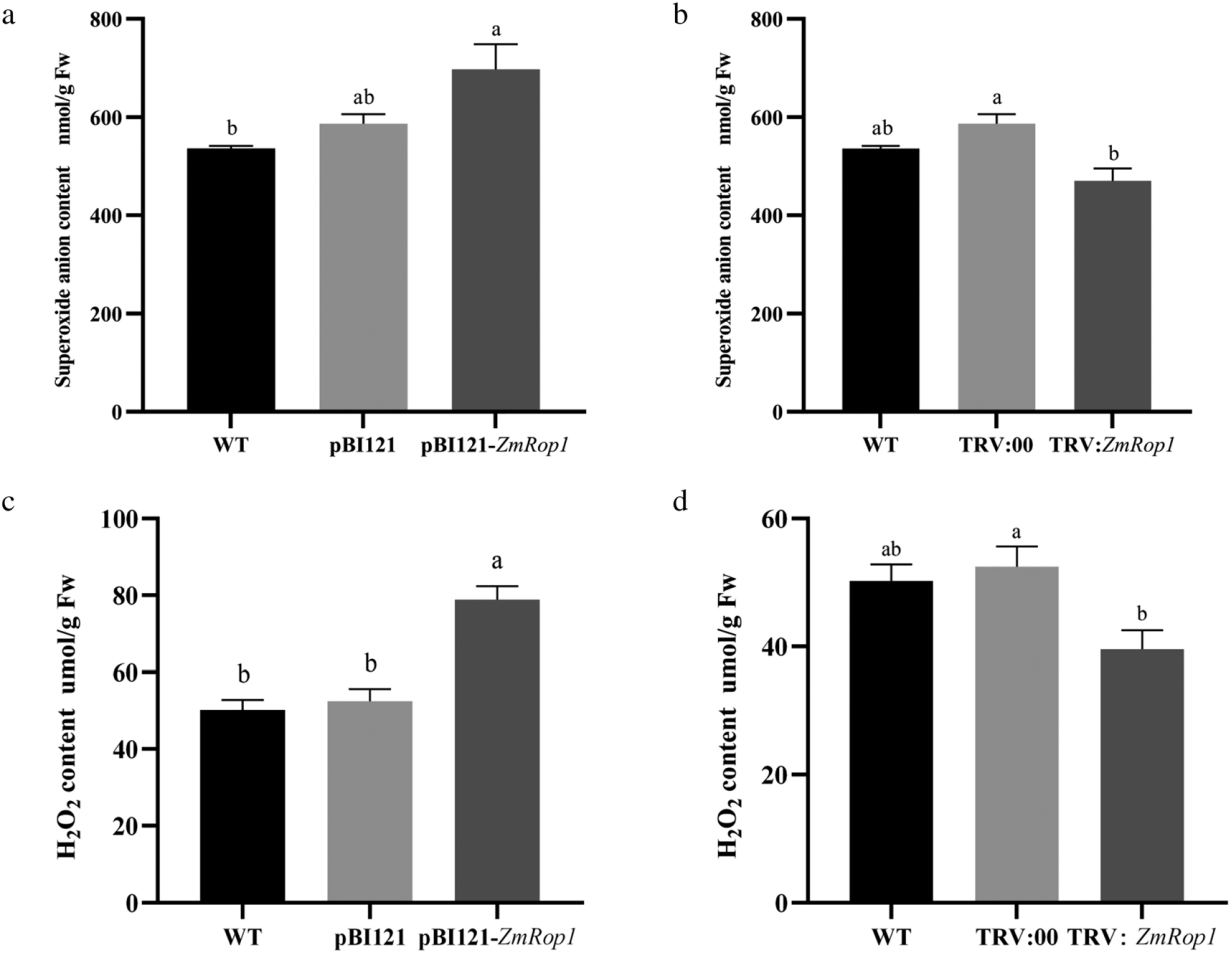
O^2−^ and H_2_O_2_ content in pBI121‐*ZmRop1* maize and TRV:*ZmRop1* maize. (a) The content level of O^2−^ in WT, pBI121 and pBI121‐*ZmRop1* maize plants. (b) The content level of O^2−^ in WT, TRV:00 and TRV:*ZmRop1* maize plants. (c) The content level of H_2_O_2_ in WT, pBI121 and pBI121‐*ZmRop1* maize plants. (d) The content level of H_2_O_2_ in WT, TRV:00 and TRV:*ZmRop1* maize plants. Different letters indicate significant differences (*p* < .05) based on Tukey's HSD test.

Plant Rops have functionally associated with ROS in improving plant tolerance to stress (Zhang et al., 2020). Therefore, the content changes of O^2−^ and H_2_O_2_, the main species of ROS, were studied. NBT staining results showed that more intensely stained spots appeared on pBI121‐*ZmRop1* plants compared with pBI121 and WT maize plants. By contrast, less intense staining was apparent in TRV:*ZmRop1* plants compared with TRV:00 and WT plants (Figure S3). In the subsequent quantitative determination results, the O^2−^ content in pBI121‐*ZmRop1* maize was the highest, and there was a significant difference between the O^2−^ content in pBI121‐*ZmRop1* and WT maize plants (Figure 6a). Inversely, O^2−^ content in TRV:*ZmRop1* maize plants was significantly lower than that in TRV:00 plants (Figure 6b). The content of H_2_O_2_ in pBI121‐*ZmRop1* maize plants was significantly higher than that in WT and pBI121 maize plants (Figure 6c). However, the content of H_2_O_2_ in TRV:*ZmRop1* maize plants was significantly lower than that of TRV:00 maize plants (Figure 6d). These results indicated that *ZmRop1* expression influences the generation of ROS.

### 3.7 *ZmRop1* expression influences enzyme activities and expression levels on ROS generation

**FIGURE 7:**
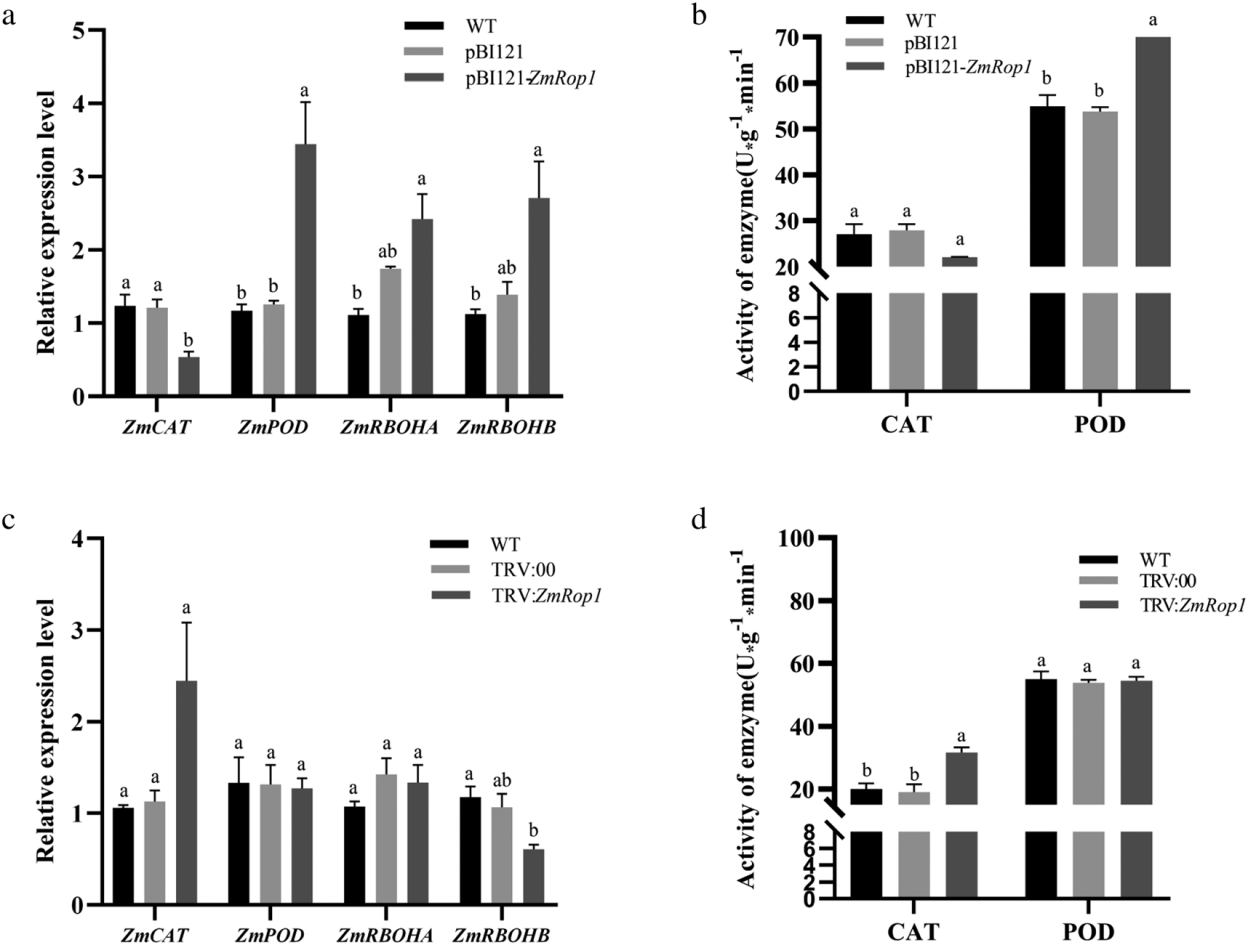
The expression level and activities of key genes and enzymes in the ROS pathway in pBI121‐*ZmRop1* and TRV:*ZmRop1* maize. (a) The expression levels of four genes (*ZmCAT*, *ZmPOD*, *ZmRBOHA*, and *ZmRBOHB*) in WT, pBI121 and pBI121‐*ZmRop1* maize plants. (b) The activities of CAT and POD in WT, pBI121 and pBI121‐*ZmRop1* maize plants. (c) Expression levels of four genes (*ZmCAT*, *ZmPOD*, *ZmRBOHA*, and *ZmRBOHB*) in WT, TRV:00 and TRV:*ZmRop1* maize plants. (d) The activities of CAT and POD in WT, TRV:00 and TRV:*ZmRop1* maize plants. Different letters indicate significant differences (*p* < .05) based on Tukey's HSD test.

It is known that the catalase (CAT), peroxidase (POD) and respiratory burst oxidase homolog (RBOH) are related to H_2_O_2_ generation. Therefore, their expression levels and enzyme activities were analyzed (Figure 7). Compared with WT and pBI121 maize plants, the expression level of *ZmCAT* in pBI121‐*ZmRop1* maize plants was significantly down‐regulated, and CAT enzyme activity also decreased but there was no significant difference (Figure 7a,b). *ZmPOD* expression level and POD enzyme activity in pBI121‐*ZmRop1* maize plants were both significantly higher than those in WT and pBI121 maize plants (Figure 7a,b). *ZmRBOHA* and *ZmRBOHB* expression levels were significantly higher in pBI121*‐ZmRop1* maize plants compared with WT maize plants, but there was no significant difference between the expression levels in pBI121 and pBI121‐*ZmRop1* maize plants (Figure 7a,b). Although there was no significant difference in the expression levels of CAT in WT and TRV silencing plants, an increase in CAT enzymatic activity was detected in TRV:*ZmRop1* plants. Furthermore, the expression levels and enzymatic activity of *ZmPOD* and *ZmRBOHA* were not altered in TRV:*ZmRop1* maize plants. A reduction in *ZmRBOHB* transcripts was detected in TRV:*ZmRop1* plants, and statistically significant compared with WT plants (Figure 7c,d).

### 3.8 Influence of *ZmRop1* expression on the content of the total soluble phenol

**FIGURE 8:**
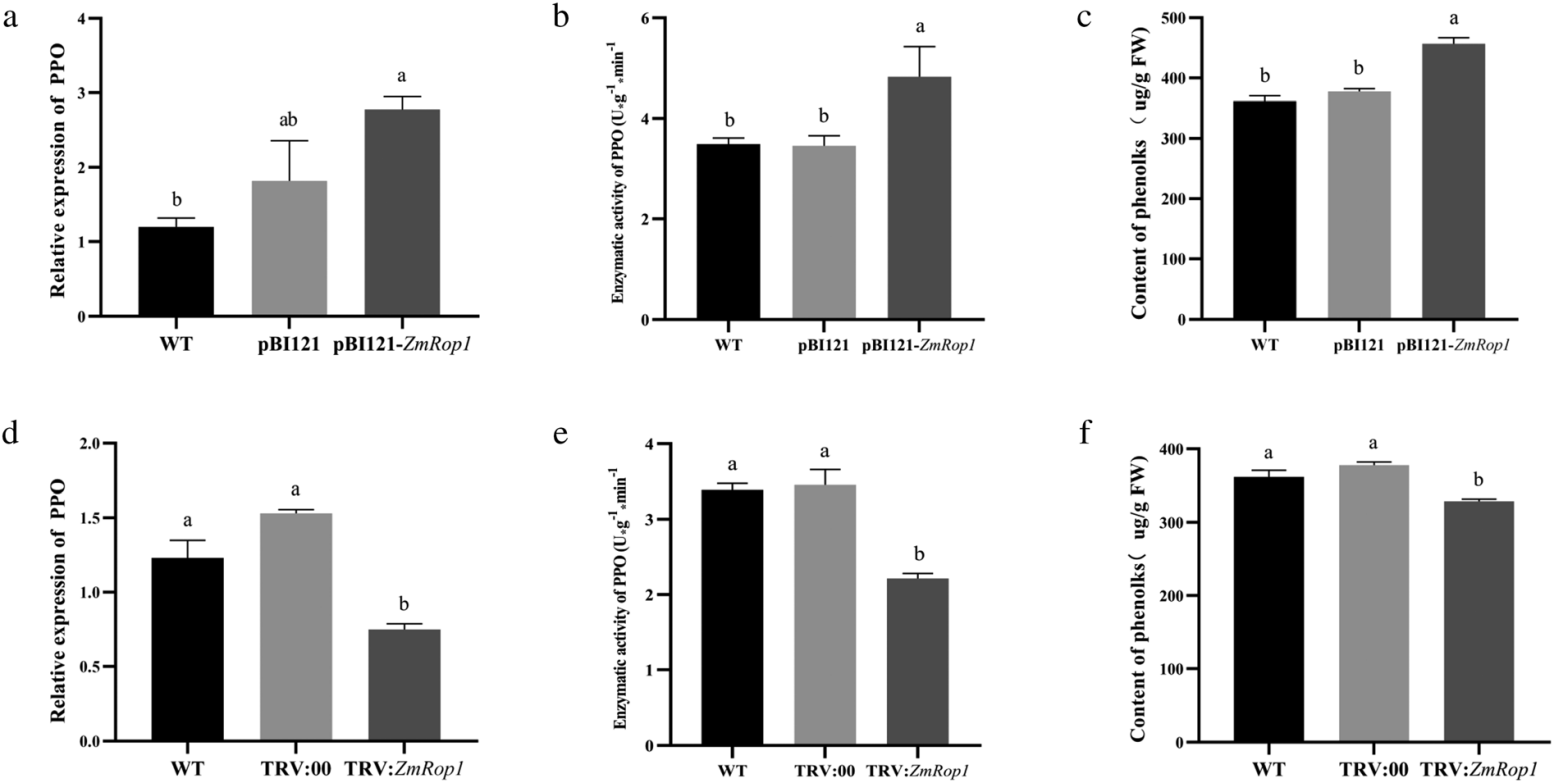
Influence of *ZmRop1* expression on the content of the phenolic compounds. (a) Expression levels of *ZmPPO* gene in WT, pBI121 and pBI121‐*ZmRop1* maize plants. (b) The activities of PPO in WT, pBI121 and pBI121‐*ZmRop1* maize plants. (c) The content of the total soluble phenol in WT, pBI121 and pBI121‐*ZmRop1* maize plants. (d) Expression levels of *ZmPPO* gene in WT, TRV:00 and TRV:*ZmRop1* maize plants. (e) Activities of PPO in WT, TRV:00 and TRV:*ZmRop1* maize plants. (f) The content of the total soluble phenol in WT, TRV:00 and TRV:*ZmRop1* maize plants. Different letters indicate significant differences (*p* < .05) based on Tukey's HSD test.

The content of the total soluble phenol is closely related to plant insect resistance, and polyphenol oxidase (PPO) is involved in plant‐insect resistance and ROS removal (Zhang & Sun, 2021). Therefore, we determined the activity and expression of PPO as well as the content of total soluble phenol. The results showed that the *ZmPPO* expression level was significantly higher in pBI121‐*ZmRop1* maize plants than in WT maize plants but incomparable to pBI121 maize plants (Figure 8a). In terms of PPO enzymatic activity and the soluble phenol content, pBI121‐*ZmRop1* maize plants exhibited the highest levels than the control plants (Figure 8b,c). The transcripts and enzymatic activity of PPO and the phenol content in TRV:*ZmRop1* maize plants were significantly lower compared with WT and TRV:00 maize plants (Figure 8d–f).

## 4 DISCUSSION

As a crucial molecular switch in plant signal transduction processes, Rops play vital roles in responses to biotic and abiotic stresses in the plant, including cytoskeletal organization, hormone responses, stress responses, and pathogen resistance (Feiquelman et al., 2018). The amino acid sequence and phylogenetic tree of nine maize Rop proteins showed that ZmRops had high homology with other plant Rop proteins, and had conserved domains of GTP, GDI, GEF and typical G‐box sequences (Figure 1). Therefore, we predicted that ZmRops may be as responsive to biological stress caused by FAW damage as ROP proteins in other model plants.

Recent studies have confirmed that *ZmRops* are closely related to maize growth and development and response to all kinds of stresses. For instance, *ZmRop1* has been found to respond to the *Sugarcane mosaic virus* and potato virus X infection, but its defense mechanism behind this phenomenon is elusive (Cao et al., 2012). Moreover, *ZmRop2* involves in a competitive advantage over male gametophyte and *ZmRop2/9* participates in maize polarized cell growth (Arthur et al., 2003; Humphries et al., 2011). However, the response of *ZmRops* to herbivorous insects has not been reported up to now (Arthur et al., 2003). FAW is one of the most devastating polyphagous herbivore and feeds on over 300 plant species. Among these, maize is the preferred host of FAW larvae which causes maize yield loss of 15%–73% (Casmuz et al., 2010; Montezano et al., 2018).

In our study, the transcripts of *ZmRop1–9* were all regulated to varying degrees at different time points upon FAW larvae infestation (Figure 2), indicating that *ZmRop*1–9 are involved in maize response to FAW larvae feeding (Yang et al., 2018). However, the expression level of *ZmRop1* in maize leaves was induced at different time points under induction of mechanic damage, but inhibited by FAW feeding (Figures 2 and 3a). Therefore, we speculated that a certain component in FAW larvae saliva might inhibit the maize *ZmRop*1‐mediated defense response to FAW larvae. In fact, similar patterns have been reported in other insects. For example, *Helicoverpa armigera's* salivary protein can inhibit mechanical injury‐induced expression of jasmonic acid signaling pathway‐related genes (Chen et al., 2019). These results arouse our greater interest in the function and mechanism of *ZmRop1* in response to FAW larvae damage. In addition, hormone induction (MeJA,SA) led to the down‐regulation of *ZmRop1* (Figure 3b,c), we assumed that the concentrations of JA or SA in plants increases after spraying the hormone, which activates the downstream defense of plants, but excessive defense responses will affect the normal growth of plants and even impeded plant growth (Ito & Sakai, 2009). Therefore, plants will block the synthesis of JA or SA and inhibit the expression of upstream signals including ROS to avoid over‐defense (Neuser et al., 2019). As an important molecular switch involved in the regulation of ROS (Zhai et al., 2021), and Rop protein might be involved in this process.

Although the mechanism of *ZmRop1*‐mediated disease resistance has not been studied, *ZmRop1* has proved to improve the resistance of maize to sugarcane mosaic virus and the tobacco resistance to potato virus X (Cao et al., 2012), therefore, we inferred that *ZmRop1* could improve the resistance of maize to FAW to a certain extent. To confirm this speculation, *ZmRop1* was overexpressed and silenced (VIGS) in maize plants, respectively (Burch‐Smith et al., 2004; Li et al., 2018). The results showed that overexpression of *ZmRop1* enhanced maize plant tolerance to 1st to 3rd instar larvae of FAW (Figure 4), whereas *ZmRop1* silence impaired maize plant tolerance to 1st to 3rd instar larvae of FAW to a certain extent (Figure 5). These results indicated that *ZmRop1* was involved in plant response to the damage of 1st to 3rd instar larvae of FAW and were similar to other plant Rop protein functions which have been confirmed to increase plant resistance to *Myzus persicae* and *Aphis gossypii* Glover (Qiu et al., 2016; Yang et al., 2018).

ROS refer to any oxygen derivative that is more reactive than an oxygen molecule (O_2_) itself (Mittler, 2017). Among them, O^2−^ and H_2_O_2_ are the most abundant ROS in cells and key signal molecules for plant growth and development and resistance to all kinds of stresses (Mhamdi & Van Breusegem, 2018). Our results showed that the contents of O^2−^ and H_2_O_2_ in pBI121‐*ZmRop1* plants were higher than those in WT and pBI121 control. These substances were just the opposite in TRV silencing plants (Figure 6). The current studies have shown that ROS and reductant/oxidant (redox) signaling play central roles in plant‐insect interactions under herbivore attack (Kerchev et al., 2012). Rice planthoppers can secrete vitellogenin (Vg) to inhibit the accumulation of H_2_O_2_ in plants, which in turn improved insect feeding performance. Silencing Vg reduced insect feeding and survival on rice (Ji et al., 2021). However, ROS acts as a double‐edged sword. Continuously increasing ROS levels cause oxidative stress, which ultimately adversely affect plants (Reyt et al., 2015). Plants must remove excessive ROS through enzyme systems and non‐enzymatic compounds. The enzyme systems mainly have POD and CAT that are closely relative to plant insect resistance (Huang et al., 2015). POD and CAT catalyze the decomposition of H_2_O_2_ into O_2_ and H_2_O, POD also has the effect of eliminating phenolic toxicity (Huang et al., 2014). In our study, some genes and enzymes related to ROS were also basically consistent with this result, although some data were not significantly different from the control group, especially when *ZmRop1* was silenced (Figure 7). We hypothesized that other Rop proteins in maize may play a role, because different Rop proteins in the same host plant can also show diverse or even opposite functions. For example, Chen et al. (2010) demonstrated that *OsRac1* positively regulated blast resistance in rice, whereas *OsRac4* and *OsRac5* negatively regulated blast resistance in rice. In addition, overexpression of *ZmRop1* resulted in down‐regulation of CAT and up‐regulation of POD at the transcripts levels and enzyme activities (Figure 7). The phenomenon is not unique, in the study of Chan and Tian (2006), POD activity was enhanced in yeast‐treated fruit, but activity of CAT showed a decreased trend in the same fruit. To the steady‐state level of O^2−^ and H_2_O_2_, CAT and POD activities in cells are in a dynamic equilibrium, and POD also has the effect of eliminating phenolic toxicity (Chan & Tian, 2006; Wang et al., 2005). Therefore, our results suggest that *ZmRop1* is involved in maize defense to the damage of FAW through mediating the generation of ROS.

Furthermore, plant phenolic substances such as catechins and tannins in plants can inhibit or poison insects (Chalker‐Scott & Fuchigami, 2018). In this study, overexpression of *ZmRop1* significantly increased the content of phenolic substances in maize plants, whereas silencing *ZmRop1* significantly down‐regulated them (Figure 8). In addition, plant PPOs are ubiquitous copper metalloenzymes that oxidize phenols or polyphenols to form corresponding quinones that are important in plant response to the infestation of herbivores and infection of pathogens, and PPOs are also involved in ROS removal (Zhang & Sun, 2021). As for the regulation of RAC family genes on phenolic substances, Ma et al. (2017) proposed that wheat ROP protein enhanced resistance to tobacco blackstalk and bacterial wilt through lignin metabolism. Lignin is a complex phenolic polymer formed by three kinds of alcohol monomers, which also indirect supports that ROP gene is involved in regulating the synthesis of phenolic substances (Bhuiyan et al., 2009). These results suggest that *ZmRop1* affects PPO activity and total soluble phenol production to defend against FAW feeding.

## 5 CONCLUSIONS

In this study, we have studied the gene characteristics of *ZmRops* and functions of *ZmRop1* in maize response to FAW larvae damage. The results showed that the expression of *ZmRop1* was significantly inhibited after being FAW feeding, but significantly upregulated by mechanical injury in maize plants. Its expression was also significantly inhibited by MeJA and SA treatment. Overexpression of *ZmRop1* in maize plants decreased feeding and oviposition preference, and negatively affected the growth rate and weight gain of FAW. Conversely, silencing of *ZmRop1* increased maize plant susceptibility to FAW damage. By analyzing the potential anti‐herbivore metabolites, our results showed that *ZmRop1* modulated the enzymatic activities of CAT, POD and the expression levels of *ZmCAT*, *ZmPOD*, *ZmRBOHA* and *ZmRBOHB*, thereby enhancing the ROS production. In addition, *ZmRop1* could also influence the content of the total soluble phenol through mediating the activity of PPO. Taken together, these findings illuminated how *ZmRop1* participate in maize defense response to FAW damage as a positive regulator.

## AUTHOR CONTRIBUTIONS

Haoran Zhang: designed the study, performed most of the experiments, wrote the manuscript. Zongwei Hu: analyzed the data, wrote and revised the manuscript. Xincheng Luo: performed parts of some experiments. Yuxue Wang: performed parts of some experiments. Yi Wang: revised the manuscript. Ting Liu: interpreted the results. Yi Zhang: interpreted the results. Longyan Chu: provided helpful comments and discussions. Xiangping Wang: provided helpful comments and discussions. Yonghao Yu: conceived and designed the research. Jianmin Zhang: designed the experiment, wrote and revised the manuscript.

## CONFLICTS OF INTEREST

The authors declare that they have no known competing financial interests or personal relationships that could have appeared to influence the work reported in this paper.

## Supporting information


**Figure S1** Domain analysis of *ZmRop1*. Domain of *ZmRop1* from NCBI.

**Figure S2.** Detection of maize infection by *A. tumefaciens* . (**A**) Expression levels of *ZmRop1* at the V3 developmental stage of pBI121‐*ZmRop1*, pBI121 and WT maize plants. (**B**) Expression levels of *ZmRop1* at the V3 developmental stage of TRV:*ZmRop1*, TRV:00 and WT maize plants. Different letters indicate significant differences (P < .05) based on Tukey's HSD test.

**Figure S3.** O^2−^ deposition in pBI121‐*ZmRop1* and TRV:*ZmRop1* maize. O^2−^ levels were detected by NBT staining in WT maize plants, pBI121 maize plants, pBI121‐*ZmRop1* maize plants, TRV:00 maize plant and TRV:*ZmRop1* maize plants.

**Table S1** Primer sequences.
