# Supplementary figures and images for: ZmRop1 participates in maize defense response to the damage of Spodoptera frugiperda larvae through mediating ROS and soluble phenol production
Source: Plant Direct. 2022 Dec 15;6(12):e468. doi: 10.1002/pld3.468 (PMC9751866; doi:10.1002/pld3.468)

## Slide 1
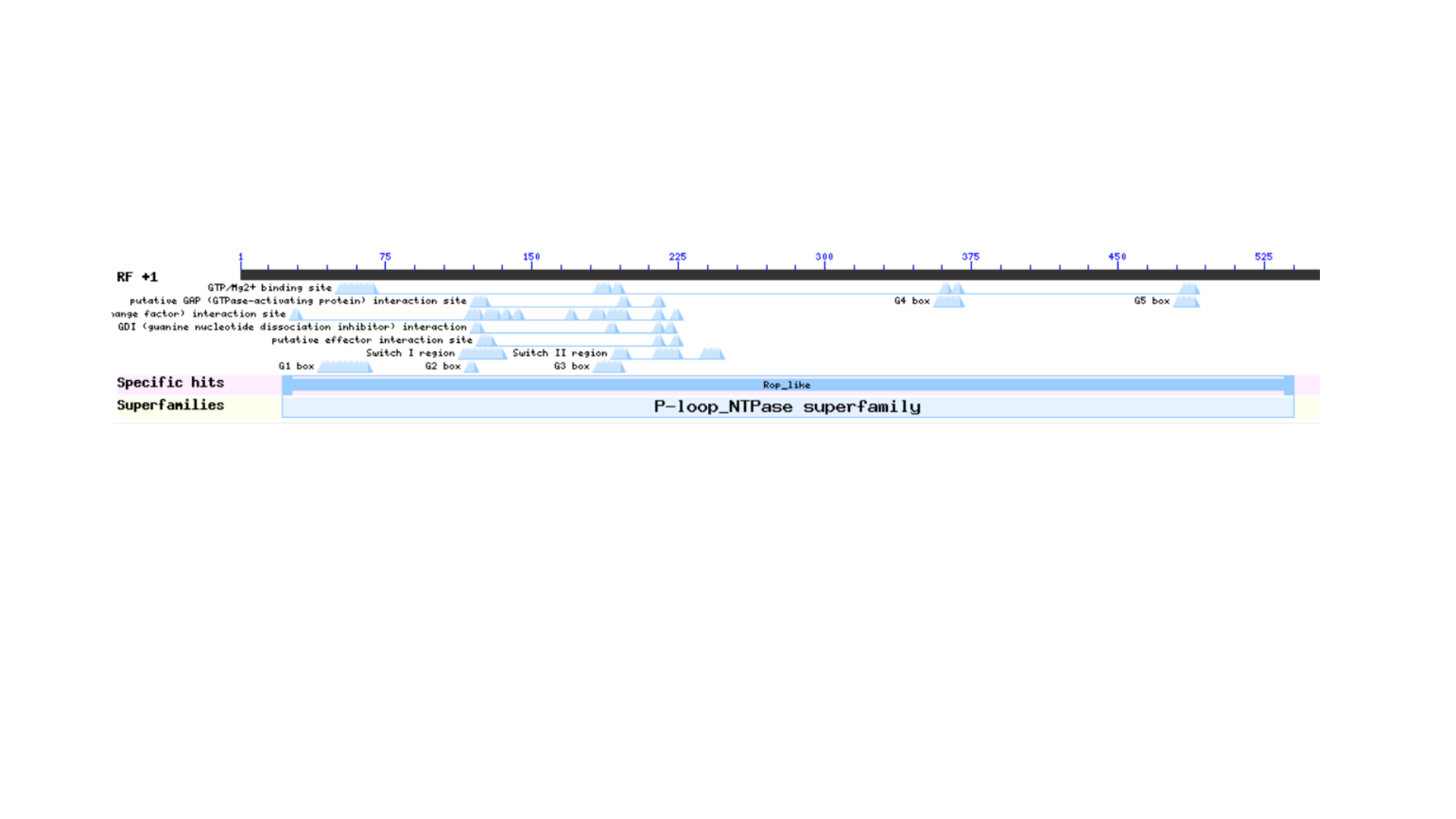

Supplement: Supplementary file 1 — Figure S1 Domain analysis of ZmRop1. Domain of ZmRop1 from NCBI. [file PLD3-6-e468-s001.pptx]

## Slide 1
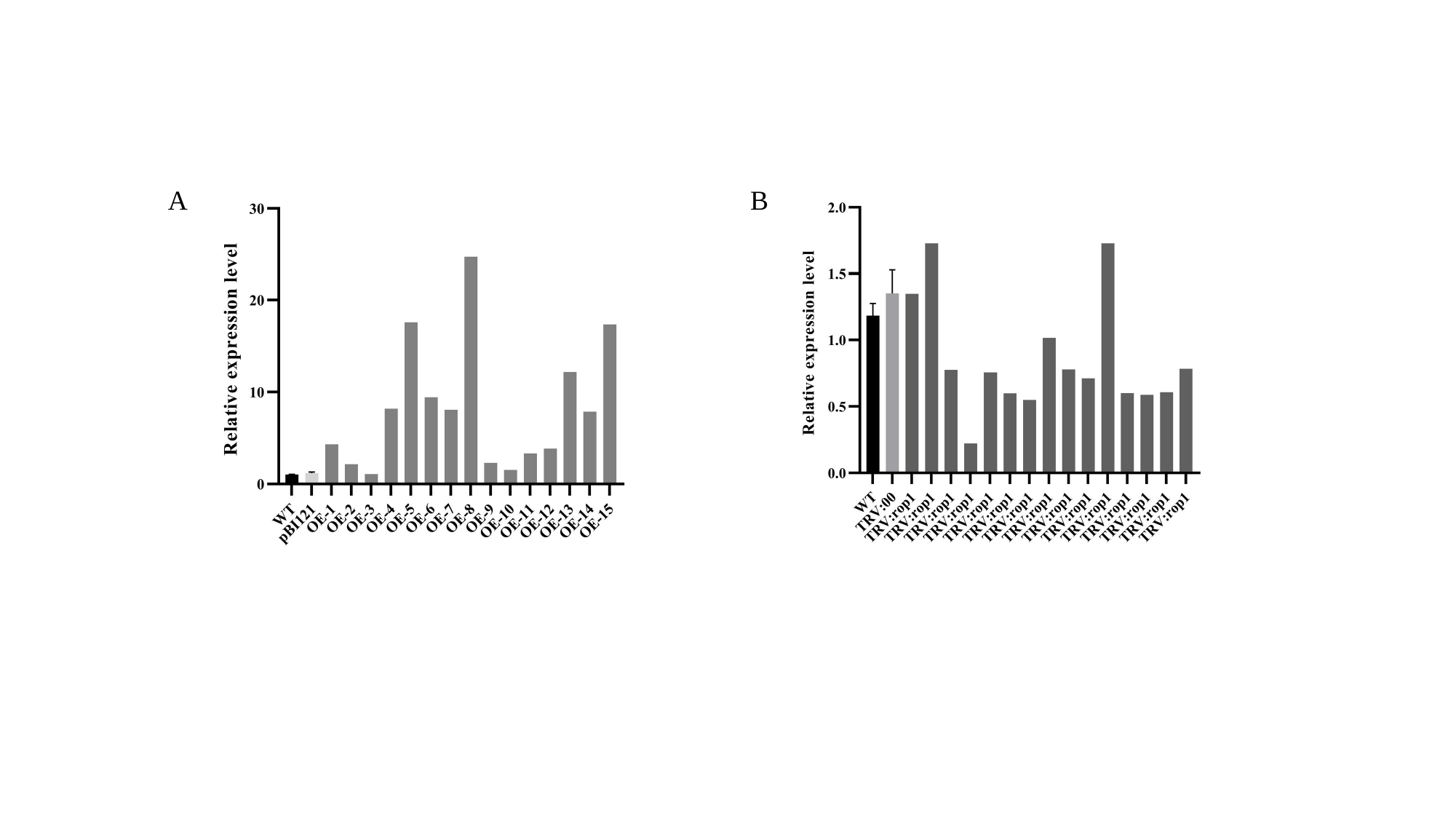

A
B

Supplement: Supplementary file 2 — Figure S2. Detection of maize infection by A. tumefaciens . (A) Expression levels of ZmRop1 at the V3 developmental stage of pBI121‐ZmRop1, pBI121 and WT maize plants. (B) Expression levels of ZmRop1 at the V3 developmental stage of TRV:ZmRop1, TRV:00 and WT maize plants. Different letters indicate significant differences (P < .05) based on Tukey's HSD test. [file PLD3-6-e468-s003.pptx]

## Slide 1
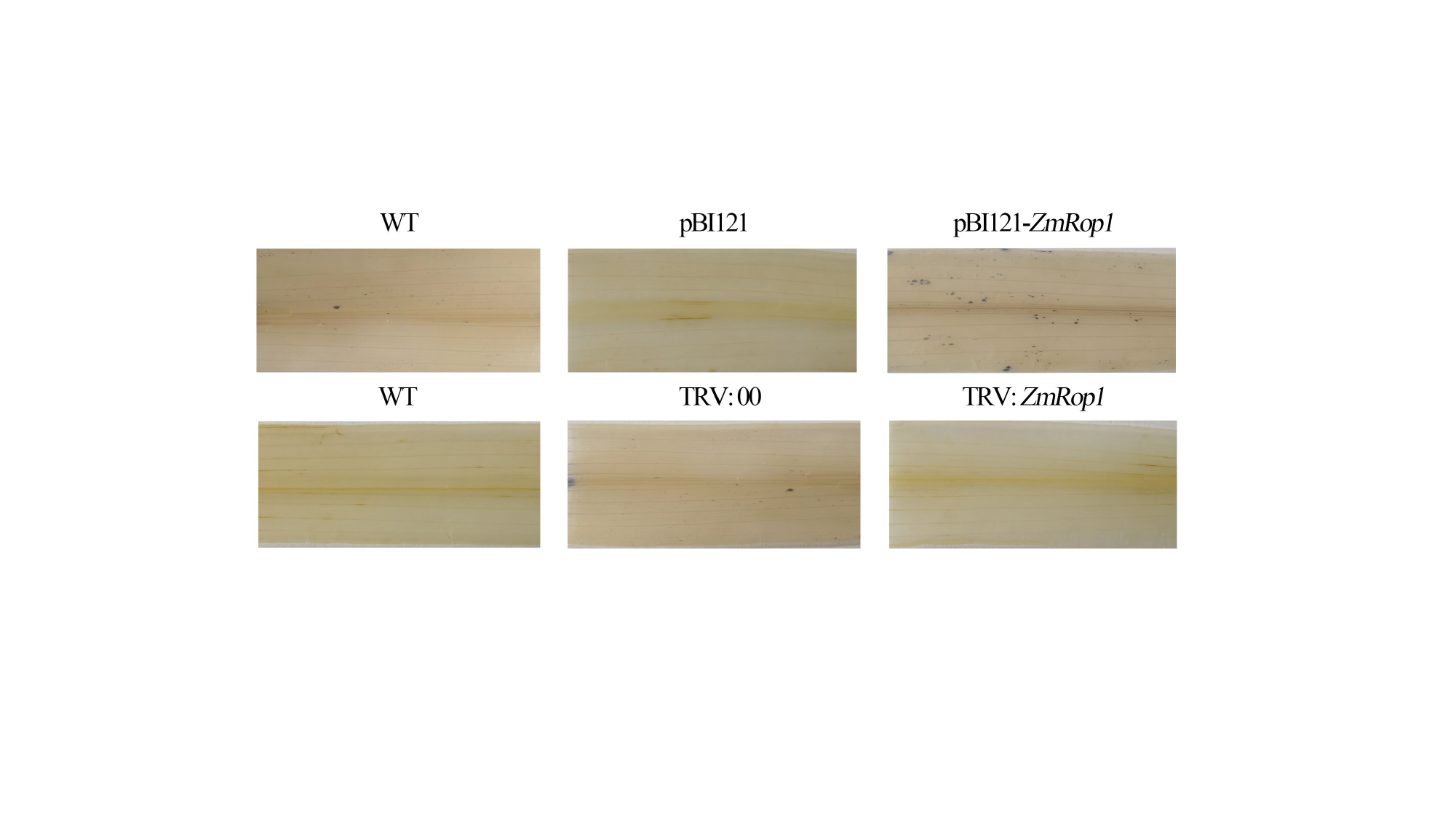

Supplement: Supplementary file 3 — Figure S3. O2− deposition in pBI121‐ZmRop1 and TRV:ZmRop1 maize. O2− levels were detected by NBT staining in WT maize plants, pBI121 maize plants, pBI121‐ZmRop1 maize plants, TRV:00 maize plant and TRV:ZmRop1 maize plants. [file PLD3-6-e468-s004.pptx]
