# Supplementary material for: ZmRop1 participates in maize defense response to the damage of Spodoptera frugiperda larvae through mediating ROS and soluble phenol production
Source: Plant Direct. 2022 Dec 15;6(12):e468. doi: 10.1002/pld3.468 (PMC9751866; doi:10.1002/pld3.468)
Supplement: Supplementary file 4 — Table S1 Primer sequences. [file PLD3-6-e468-s002.pptx]

## Slide 1
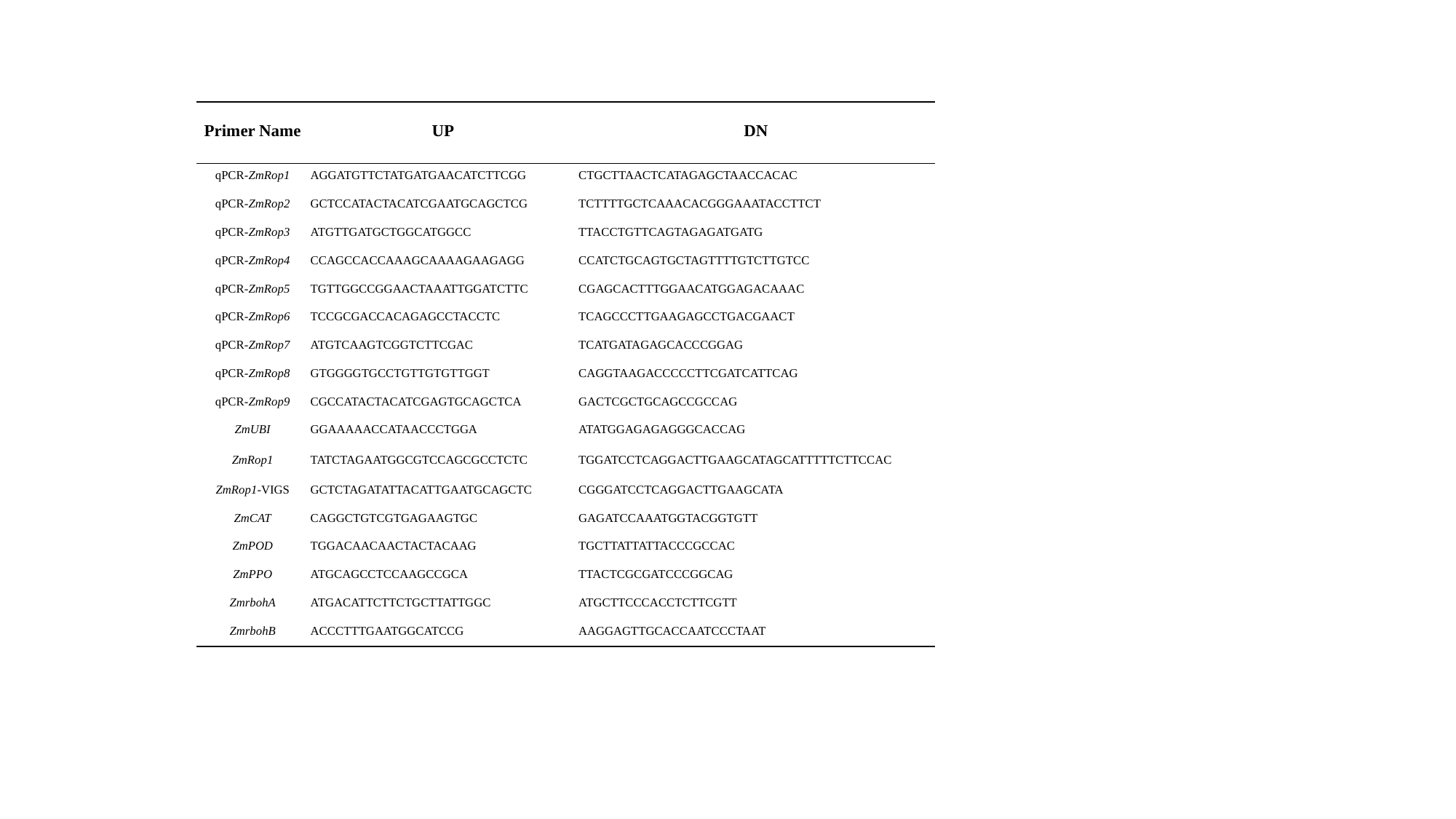

| Primer Name | UP | DN |
| --- | --- | --- |
| qPCR-ZmRop1 | AGGATGTTCTATGATGAACATCTTCGG | CTGCTTAACTCATAGAGCTAACCACAC |
| qPCR-ZmRop2 | GCTCCATACTACATCGAATGCAGCTCG | TCTTTTGCTCAAACACGGGAAATACCTTCT |
| qPCR-ZmRop3 | ATGTTGATGCTGGCATGGCC | TTACCTGTTCAGTAGAGATGATG |
| qPCR-ZmRop4 | CCAGCCACCAAAGCAAAAGAAGAGG | CCATCTGCAGTGCTAGTTTTGTCTTGTCC |
| qPCR-ZmRop5 | TGTTGGCCGGAACTAAATTGGATCTTC | CGAGCACTTTGGAACATGGAGACAAAC |
| qPCR-ZmRop6 | TCCGCGACCACAGAGCCTACCTC | TCAGCCCTTGAAGAGCCTGACGAACT |
| qPCR-ZmRop7 | ATGTCAAGTCGGTCTTCGAC | TCATGATAGAGCACCCGGAG |
| qPCR-ZmRop8 | GTGGGGTGCCTGTTGTGTTGGT | CAGGTAAGACCCCCTTCGATCATTCAG |
| qPCR-ZmRop9 | CGCCATACTACATCGAGTGCAGCTCA | GACTCGCTGCAGCCGCCAG |
| ZmUBI | GGAAAAACCATAACCCTGGA | ATATGGAGAGAGGGCACCAG |
| ZmRop1 | TATCTAGAATGGCGTCCAGCGCCTCTC | TGGATCCTCAGGACTTGAAGCATAGCATTTTTCTTCCAC |
| ZmRop1-VIGS | GCTCTAGATATTACATTGAATGCAGCTC | CGGGATCCTCAGGACTTGAAGCATA |
| ZmCAT | CAGGCTGTCGTGAGAAGTGC | GAGATCCAAATGGTACGGTGTT |
| ZmPOD | TGGACAACAACTACTACAAG | TGCTTATTATTACCCGCCAC |
| ZmPPO | ATGCAGCCTCCAAGCCGCA | TTACTCGCGATCCCGGCAG |
| ZmrbohA | ATGACATTCTTCTGCTTATTGGC | ATGCTTCCCACCTCTTCGTT |
| ZmrbohB | ACCCTTTGAATGGCATCCG | AAGGAGTTGCACCAATCCCTAAT |
